# Modulation of Adipocytokines Production and Serum NEFA Level by Metformin, Glimepiride, and Sitagliptin in HFD/STZ Diabetic Rats

**DOI:** 10.1155/2015/138134

**Published:** 2015-03-01

**Authors:** Mohamed I. Saad, Maher A. Kamel, Mervat Y. Hanafi

**Affiliations:** Biochemistry Department, Medical Research Institute, Alexandria University, 165 Elhorreya Avenue, P.O. Box 21561, Alexandria, Egypt

## Abstract

Type 2 diabetes mellitus (T2DM) is a group of metabolic disorders characterized by hyperglycemia owing to insulin resistance and/or insulin deficiency. Current theories of T2DM pathophysiology include a decline in *β*-cells function, a defect in insulin signaling pathways, and a dysregulation of secretory function of adipocytes. This study aimed to investigate the effect of different antidiabetic drugs on serum levels of certain adipocytokines and nonesterified fatty acids (NEFA) in high-fat diet (HFD)/streptozotocin- (STZ-) induced diabetic rats. All treatments significantly decreased serum NEFA level. Metformin and sitagliptin increased serum adiponectin level, whereas they decreased serum leptin level. Glimepiride showed significant decline in serum levels of both adiponectin and leptin. All treatments remarkably ameliorated insulin resistance, suggested by an improvement of glycemic control, a significant reduction in homeostasis model assessment of insulin resistance (HOMA-IR), and a correction in lipid profile. Modulation of adipocytokines production (i.e., increased serum adiponectin and decreased serum leptin) may also underlie the improvement of insulin resistance and could be a possible mechanism for the beneficial cardiovascular effects of metformin and sitagliptin.

## 1. Introduction

Type 2 diabetes mellitus (T2DM) is a progressive multifactorial, complex group of metabolic disorders characterized by hyperglycemia owing to impaired insulin sensitivity and/or insulin deficiency. T2DM is a multiorgan disease which affects multiple organs or tissues. Current theories of T2DM pathophysiology include a decline in the pancreatic *β*-cells function, a defect in insulin-mediated glucose uptake in muscles, a defect in intracellular insulin signaling pathways, a dysregulation of secretory function of adipocytes, and an impaired insulin action in liver and other organs [1]. Disrupted metabolism in T2DM causes extensive microvascular and macrovascular complications responsible of its elevated morbidity and reduced life expectancy [1]. It was estimated that 285 million people had diabetes mellitus worldwide in 2010, and this figure is projected to grow to 439 million by 2030 [2].

Adipose tissue plays an integral role in developing insulin resistance and T2DM. Resistance of adipose tissue to the antilipolytic effect of insulin results in extensive release of nonesterified fatty acids (NEFA) into blood circulation. Elevated NEFA concentrations exacerbate insulin resistance by diminishing insulin-stimulated glucose intake in muscles, directly affecting insulin signaling, activating gluconeogenesis and triglyceride synthesis in liver, and contributing to *β*-cells failure [3].

In addition to its role in fat storage, adipose tissue is now considered an active endocrine organ which secretes a wide range of bioactive factors, collectively termed as adipocytokines or adipokines (e.g., adiponectin and leptin). Adipocytokines play important roles in appetite and satiety, fat distribution, insulin secretion and sensitivity, blood pressure, endothelial function, and inflammatory reactions [4]. Adipose tissue dysfunction causes dysregulated production or secretion of these adipocytokines which is attributable to the pathogenesis of insulin resistance, T2DM, and obesity-related complications [5].

Adiponectin is a protein which generally exerts insulin-sensitizing, anti-inflammatory, antiapoptotic, antiatherogenic, and antithrombotic actions [6]. The effects of adiponectin are mediated through two receptors: adiponectin receptor 1 (AdipoR1) and adiponectin receptor 2 (AdipoR2) [7]. Leptin is an anorexia peptide which modulates body weight, energy expenditure, food intake, and fat stores through its action on the hypothalamus [6]. Leptin exerts its actions via binding to leptin receptors; the Ob/Rb is the best characterized leptin receptor [8]. Interestingly, insulin resistance is associated with decreased levels of adiponectin and increased levels of leptin, reflecting a state of adiponectin deficiency and leptin resistance [9]. Consequently, some adipocytokines are considered as innovative biomarkers for diagnosis and therapeutic monitoring of insulin resistance, obesity, and T2DM [4]. Therefore, treatments that regulate the production or secretion of adipocytokines might be a promising way for the prevention of obesity-related metabolic and cardiovascular disorders.

The purpose of this study was to investigate the effect of different antidiabetic drugs (metformin, glimepiride, and sitagliptin) on serum levels of certain adipocytokines (adiponectin and leptin) and NEFA. We also carried out routine biochemical analysis to assess glucose homeostasis parameters (e.g., blood glucose level, serum insulin, and insulin resistance using the homeostasis model assessment) and lipid profile. This study was performed on high-fat diet (HFD)/streptozotocin- (STZ-) induced diabetic rats.

## 2. Materials and Methods

### 2.1. Experimental Animals

Wistar rats aged 8 weeks were obtained from the Medical Technology Center (Alexandria, Egypt). The rats were housed 4 per cage at an ambient temperature of 23 ± 1°C with 12/12 h light/dark cycles and 45 ± 5% humidity. Rats had free access to chow diet and water for a week prior to the experiment. The study was done in accordance with the ethical guidelines of the Medical Research Institute, Alexandria University, Egypt.

### 2.2. Experimental Design

The experimental animals were divided into five groups, each group consisting of 10 rats detailed as follows. Group (1) served as the normal control rats and were administered dimethyl sulfoxide (DMSO) as a vehicle without any treatments. The rest of animals were rendered diabetic by feeding a high-fat diet containing 40% fats (HFD) for 4 weeks, followed by a single intraperitoneal injection of STZ at a low dose (45 mg/kg of body weight, dissolved in 0.05 M citrate buffer, pH 4.5, immediately before use). One week after injection, fasting blood glucose (FBG) levels were determined from tail blood using an Accu-Chek Active glucometer (Roche Diagnostics, Manheim, Germany). The rats with FBG levels above 200 mg/dL were considered as diabetic [10]. Group (2) served as the diabetic untreated rats and were administered DMSO as a vehicle without any treatments. Group (3) served as diabetic rats treated with metformin (200 mg/kg of body weight). Group (4) served as diabetic rats treated with glimepiride (0.1 mg/kg of body weight). Group (5) served as diabetic rats treated with sitagliptin (10 mg/kg of body weight). Treatments were administered daily in DMSO suspension by oral gavage for 4 weeks. The dosage was adjusted every week, according to any change in body weight to maintain similar dose per kg body weight of rat over the whole period of study for each group. FBG level was measured every week. At the end of the treatment period, the rats were fasted overnight, anaesthetized with diethyl ether, and sacrificed by cervical decapitation. The blood was collected for serum separation and biochemical analysis [10].

### 2.3. Biochemical Analysis

To assess oral glucose tolerance at the end of the treatment period (4 weeks), animals were fasted overnight for 12 hours and their serum glucose response to the oral administration (by gavage) of a solution of glucose (2.5 g/kg of body weight) was determined. Tail blood samples were taken before time 0 and 30, 60, 90, and 120 minutes after administration of glucose, and glucose level was determined with an Accu-Chek Active glucometer [10].

The level of serum glucose was estimated using an Accu-Chek Active glucometer. Serum insulin level was assayed using a sandwich ELISA kit (Millipore) according to the manufacturer's instructions. The insulin resistance index (IRI) was assessed by homeostasis model assessment estimate of insulin resistance (HOMA-IR) as follows:

(1) IRI=Fasting  insulinµIU/mL×Fasting  glucosemmol/L22.5.

Lipid profile was assessed by using a commercial diagnostic kit (Randox (UK)) according to the manufacturer's instructions. Levels of adiponectin, leptin, and NEFA in rat serum were assessed using ELISA kits (Chemicon, RayBio, and MyBioSource, resp.) according to the manufacturer's instructions.

### 2.4. Statistical Analysis

The data were analyzed using the one-way analysis of variance (ANOVA) followed by LSD test to compare different groups with each other (SPSS software). Results were expressed as mean ± standard deviation (SD) and values of *P* > 0.05 were considered nonsignificantly different, while those of *P* < 0.05 were considered significant.

## 3. Results

### 3.1. Body Weight Change

After induction of diabetes and before starting the treatments, all diabetic rats were obese and showed significant increase in body weight compared to the control group (Table 1). At the end of the treatment period, all diabetic groups (treated and untreated) showed no significant difference from the control one (Table 1).

### 3.2. Glucose Homeostasis Parameters

After induction of diabetes and before the administration of treatments, all diabetic rats showed significantly elevated levels of FBG (hyperglycemia) compared to the control rats (Table 2). All treated rats showed a time-dependent reduction in FBG during the treatment period. During this time period, it was apparent that all treated rats had significantly lower FBG than the untreated rats. By the end of the treatment period (4 weeks), FBG levels were normalized by all drug treatments (Table 3).

Before starting the treatments, all diabetic rats showed higher serum insulin levels (hyperinsulinemia) compared to the control rats (Table 2). By the end of the treatment period, all diabetic groups (untreated and treated) exhibited a significantly higher serum insulin levels compared to the control rats. Moreover, there are no significant variations in the serum insulin levels in different diabetic treated rats compared with those of untreated ones (Table 3).

The insulin resistance index calculated by the HOMA model (HOMA-IR) using the level of fasting insulin (*µ*IU/mL) and glucose level (mmol/L) indicated that all diabetic rats started the study with significantly higher HOMA-IR values compared to the control rats (Table 2). At the end of the treatment period, all of the treated rats showed a significant decline in the insulin resistance index compared to the untreated rats with the least value observed in rats treated with metformin (Table 3).

**Figure 1:**
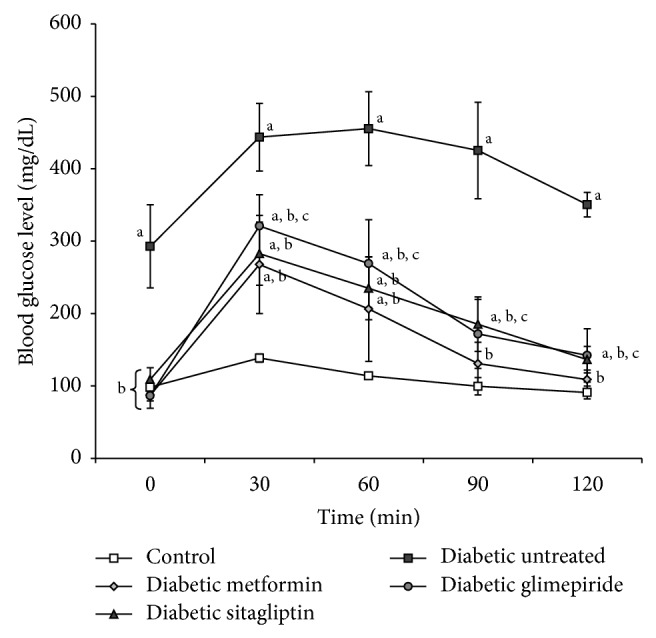
Change in blood glucose level (mg/dL) during the oral glucose tolerance test (OGTT) of different studied groups done at the end of the treatment period (4 weeks). a: significantly different from the control group, b: significantly different from the diabetic untreated group, and c: significantly different from the Metformin group, using ANOVA (LSD), *P* value < 0.05.

From the OGTT performed at the end of treatment period, it was obvious that the untreated diabetic rats were suffering from impaired fasting glucose tolerance and impaired glucose tolerance during the two-hour period of the test (Figure 1), while the treated diabetic rats showed no impairment in the fasting glucose tolerance but showed impaired glucose tolerance after glucose administration which was of lesser extent than that observed in the untreated rats (Figure 1). The best response in the OGTT was associated with metformin treatment followed by sitagliptin treatment and the least response was observed with glimepiride treatment (Figure 1).

### 3.3. Lipid Profile

The baseline values of the lipid profile (triglycerides, total cholesterol, HDL-C, and LDL-C) showed significantly higher levels of triglycerides, cholesterol, and LDL-C and lower level of HDL-C in the untreated diabetic rats than the control rats (Table 2).

**Figure 2:**
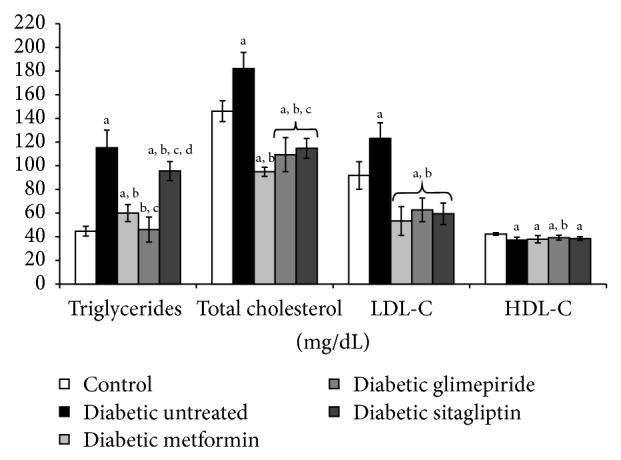
Lipid profile of different studied groups at the end of the treatment period (4 weeks). a: significantly different from the control group, b: significantly different from the diabetic untreated group, c: significantly different from the Metformin group, and d: significantly different from the Glimepiride group, using ANOVA (LSD), *P* value < 0.05.

At the end of the treatment period, metformin and glimepiride significantly corrected the levels of triglyceride, cholesterol, and LDL-C (Figure 2). Sitagliptin treatment slightly but significantly decreased the level of triglycerides and completely normalized the levels of cholesterol and LDL-C. All treatments at the administered doses showed no or slight effect on the HDL-C level (Figure 2).

### 3.4. Serum Level of NEFA

**Figure 3:**
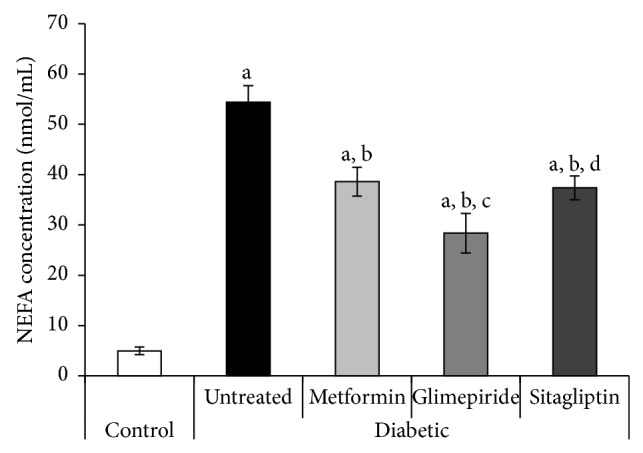
Serum nonesterified fatty acids (NEFA) level (mmol/mL) of different studied groups at the end of the treatment period (4 weeks). a: significantly different from the control group, b: significantly different from the diabetic untreated group, c: significantly different from the Metformin group, and d: significantly different from the Glimepiride group, using ANOVA (LSD), *P* value < 0.05.

At the end of the experiment, the untreated diabetic rats showed great elevation in serum NEFA levels to be 10.9-fold the control value (54.4 ± 3.3 and 4.99 ± 0.8 nmol/mL, resp.) (Figure 3). All treatments significantly decreased serum NEFA level compared with the diabetic untreated rats and the least NEFA level was observed in glimepiride-treated rats (Figure 3).

### 3.5. Serum Levels of Adipocytokines

**Figure 4:**
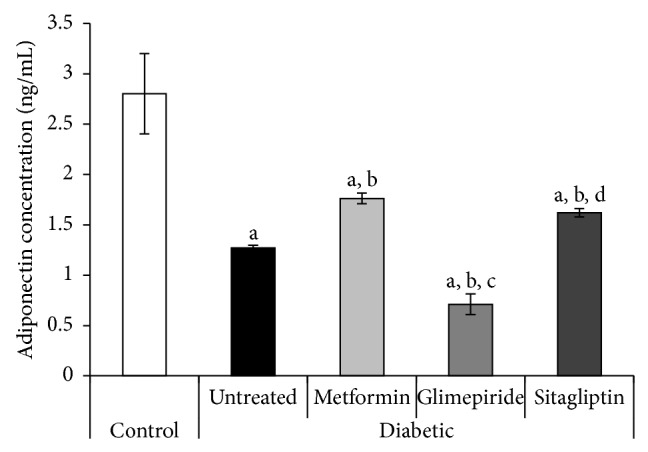
Serum adiponectin level (ng/mL) of different studied groups at the end of the treatment period (4 weeks). a: significantly different from the control group, b: significantly different from the diabetic untreated group, c: significantly different from the Metformin group, and d: significantly different from the Glimepiride group, using ANOVA (LSD), *P* value < 0.05.

**Figure 5:**
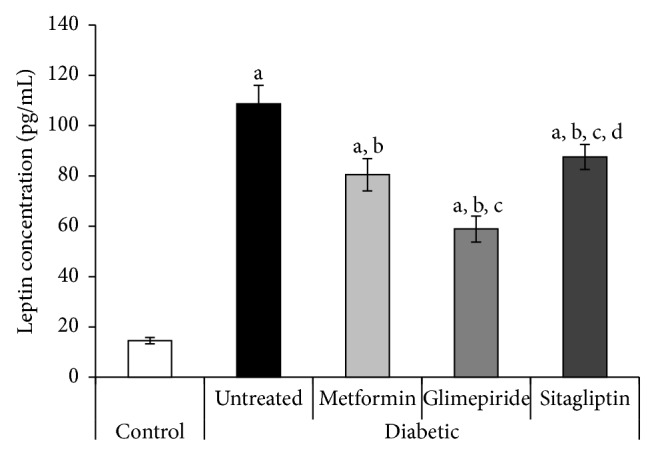
Serum leptin level (pg/mL) of different studied groups at the end of the treatment period (4 weeks). a: significantly different from the control group, b: significantly different from the diabetic untreated group, c: significantly different from the Metformin group, and d: significantly different from the Glimepiride group, using ANOVA (LSD), *P* value < 0.05.

At the end of the experiment, the untreated diabetic rats showed a great decline in serum adiponectin level to be 0.4-fold the control value (1.27 ± 0.03 and 2.8 ± 0.4 ng/mL, resp.) (Figure 4). On the contrary, the same rats showed a great elevation in serum leptin level to be 7.5-fold the control value (108.7 ± 7.3 and 14.5 ± 1.3 pg/mL, resp.) (Figure 5).

Compared with the diabetic untreated group, metformin and sitagliptin at the administered doses significantly increased serum adiponectin level, whereas they decreased serum leptin level (Figures 4 and 5). Furthermore, glimepiride-treated rats showed significant decline in serum levels of both adiponectin and leptin to be the least values observed compared to all the other diabetic groups (treated and untreated) (Figures 4 and 5).

## 4. Discussion

The current study was designed to demonstrate the effects of the antidiabetic drugs metformin, glimepiride, and sitagliptin on serum levels of adiponectin, leptin, and NEFA as well as glucose homeostasis parameters and lipid profile. Induction of diabetes was done by feeding the rats HFD for 4 weeks followed by a single I.P. injection of STZ at 45 mg/kg of body weight. HFD induces insulin resistance, while the low dose of STZ results in mild dysfunction of *β*-cell function. This HFD/STZ diabetic model replicates the metabolic characteristics of T2DM indicated by overt hyperglycemia, dyslipidemia, obesity, hyperinsulinemia, and insulin resistance (as shown by increased HOMA) [10]. With respect to body weight at the end of the study, although metformin administration is associated with weight loss, all administered drugs at the assigned doses were proved to be weight-neutral agents.

It was obvious that our model of T2DM exhibited elevated insulin level which could be a result of insulin resistance in peripheral tissues. Consequently, insulin was unable to act properly on resistant tissues and this resulted in poor glucose disposal and utilization; therefore, compensatory hyperinsulinemia due to enhanced *β*-cell secretion was an obligate accompanying feature in insulin resistance. Furthermore, there is no absolute definition of hyperinsulinemia, and there is no specific cut-off value at which resistance begins and sensitivity ends [11].

The antidiabetic drugs under investigation have different modes of actions. Metformin, the first choice drug for the treatment of T2DM, is a biguanide insulin-sensitizing agent, which inhibits hepatic gluconeogenesis, enhances insulin action on glucose uptake in peripheral tissues, and decreases absorption of glucose from the intestine. This decline in hepatic energy production activates AMP-activated protein kinase (AMPK), which is a cellular metabolic sensor [12]. Glimepiride is a second generation sulphonylurea which enhances insulin secretion through binding to the sulphonylurea receptors (SUR1) on pancreatic *β*-cells and thereby causes glucose-independent closure of the ATP-sensitive K^+^ channels [13]. Glimepiride could also exert extrapancreatic effects such as improving peripheral glucose uptake, insulin-sensitizer effect, and suppression of endogenous glucose production [14]. Sitagliptin is a dipeptidyl peptidase-4 (DPP-4) inhibitor which inhibits the rapid degradation of glucagon-like peptide-1 (GLP-1) and glucose-dependent insulinotropic polypeptide (GIP). Consequently, elevation in serum levels of incretin hormones (GLP-1 and GIP) inhibits glucagon secretion and stimulates glucose-dependent insulin secretion [15].

The main purpose of the antidiabetic therapies is to reduce and maintain glucose levels as close to normal and thereby prevent the development of complications. However, individual responses to these drugs can differ greatly, probably owing to the heterogeneous nature of the pathophysiology of T2DM [16]. In our experiment, all drugs at the administered doses resulted in lowering of glucose levels and improvement of the glycemic control in diabetic rats. It was apparent that the antihyperglycemic effect of metformin was not associated with enhancing circulating insulin levels, and this is consistent with the belief that metformin significantly decreases insulin levels [12]. Moreover, all drugs remarkably improved insulin resistance, suggested by a significant reduction in HOMA values.

Our HFD/STZ model exhibited the main features of diabetic dyslipidemia: a high serum triglyceride, low HDL-cholesterol, and increased LDL-cholesterol levels. This is caused mainly by increased lipolysis (i.e., increased NEFA release) from insulin-resistant adipocytes [17]. The results of our study indicated an elevated serum NEFA level in the diabetic rats. The increased flux of NEFA directly affects insulin signalling, diminishes glucose uptake in muscle, exaggerates triglyceride synthesis, induces gluconeogenesis in the liver, and contributes to *β*-cell failure [11]. Therefore, reducing serum NEFA concentration suppresses the driving force in insulin resistance and T2DM.

In our study, it was evident that all treatments significantly decreased serum NEFA level, and the most potent effect was observed with glimepiride. Metformin and glimepiride significantly corrected the levels of triglyceride, cholesterol, and LDL-cholesterol. Sitagliptin treatment slightly but significantly decreased the level of triglycerides and completely normalized the levels of cholesterol and LDL-cholesterol. The administered doses of all drugs exhibited no or slight effect on the HDL-cholesterol level. In line with these findings, it has been demonstrated that metformin inhibits lipolysis in adipose tissue and reduces circulating NEFA. Metformin also reduces total cholesterol and LDL-cholesterol and triglycerides [18]. Moreover, sitagliptin induces modest reduction in levels of total cholesterol, LDL-cholesterol, and triglycerides [19].

It has been proven that adiponectin is underexpressed in patients with T2DM and low adiponectin levels are associated with obesity-related metabolic disorders [20]. Adiponectin protects against the development of atherosclerosis, inflammation, endothelial dysfunction, T2DM, and obesity-linked cardiovascular diseases [21]. Our results indicated that metformin and sitagliptin, but not glimepiride, at the administered doses significantly increased serum adiponectin level compared to the untreated diabetic rats.

On the other hand, T2DM is associated with elevated leptin levels owing to a state of leptin resistance (impaired leptin signalling and action) [9]. Our study demonstrated that all drugs under investigation decreased circulating leptin levels, suggesting that these drugs could enhance leptin sensitivity and correct leptin resistance in T2DM. In line with our finding, it has been shown that metformin decreases leptin concentration in obese and normal-weight healthy subjects [22].

The data of circulating adiponectin and leptin levels suggested that metformin and sitagliptin may explain the beneficial cardiovascular effects beyond their glucose-lowering effects. Metformin is proved to enhance endothelial function and reduce cardiovascular risk associated with T2DM [23]. Furthermore, DPP-4 inhibitors exert a protective effect against vascular complications through their anti-inflammatory and endothelial repair effects [24].

## 5. Conclusion

In conclusion, the drugs under investigation improved glycemic control in T2DM. These treatments remarkably ameliorated insulin resistance, suggested by a significant reduction in HOMA-IR value and a correction in lipid profile. Furthermore, modulation of adipocytokines serum concentration (i.e., elevated plasma adiponectin and decreased plasma leptin) may also underlie the improvement of insulin resistance and could be a possible mechanism for the beneficial cardiovascular effects of metformin and sitagliptin.

## Figures and Tables

**Table 1:** Change in body weight of different studied groups during the treatment period (4 weeks).

Weeks	Control	Diabetic			
		Untreated	Metformin	Glimepiride	Sitagliptin
After induction of T2DM (before starting treatments)	113.9 ± 13.9	132.2 ± 21^a^	131.2 ± 13.9^a^	130.6 ± 16.5^a^	133.3 ± 11.2^a^
Week (1)	127.8 ± 18.9	134.3 ± 23	140.4 ± 12	137 ± 13.4	135.7 ± 14.1
Week (2)	129.9 ± 17.1	145.2 ± 21.2^a^	139.1 ± 8.6	139.9 ± 17.2	140.3 ± 15.1
Week (3)	139.2 ± 19.7	149.1 ± 23.1	141.3 ± 11.7	150.9 ± 22.5	155.4 ± 15.5
Week (4)	146.1 ± 18.3	151.6 ± 25.8	142.9 ± 10.1	154.8 ± 21.5	160.6 ± 17.5^c^

Values are presented as mean ± SD (*n* = 10). ^a^Significantly different from the control group and ^c^significantly different from the Metformin group, using ANOVA (LSD), *P* value < 0.05.

**Table 2:** Baseline values of glucose homeostasis parameters and lipid profile of different studied groups.

Parameter	Control	Diabetic			
		Untreated	Metformin	Glimepiride	Sitagliptin
Fasting blood glucose (mg/dL)	88 ± 7.1	361.8 ± 43.9^a^	354.2 ± 47^a^	355.7 ± 63.1^a^	362.9 ± 52.6^a^
Serum insulin (*µ*IU/mL)	1.7 ± 0.2	11.1 ± 0.4^a^	10.9 ± 0.4^a^	11.1 ± 0.5^a^	11.2 ± 0.3^a^
HOMA-IR	0.42 ± 0.1	7.6 ± 1.1^a^	7.8 ± 1.3^a^	7.5 ± 1.2^a^	7.6 ± 1.2^a^

Triglycerides (mg/dL)	42.3 ± 3.8	111.8 ± 13.2^a^	113.6 ± 14.8^a^	112.1 ± 12.4^a^	113.1 ± 12.9^a^
Total cholesterol (mg/dL)	142.3 ± 6.7	179.8 ± 12.1^a^	178.1 ± 11.8^a^	180.1 ± 12.6^a^	179.4 ± 11.7^a^
LDL-cholesterol (mg/dL)	89.4 ± 12.8	121.8 ± 14.7^a^	122.3 ± 12.9^a^	122.5 ± 13.4^a^	121.2 ± 13.1^a^
HDL-cholesterol (mg/dL)	41.2 ± 0.9	37.1 ± 1.9^a^	37.4 ± 2.1^a^	36.9 ± 2.2^a^	37.2 ± 1.8^a^

Values are presented as mean ± SD (*n* = 10). ^a^Significantly different from the control group by ANOVA (LSD), *P* value < 0.05.

**Table 3:** Glucose homeostasis parameters of different studied groups during and at the end of the treatment period (4 weeks).

Parameter	Control	Diabetic			
		Untreated	Metformin	Glimepiride	Sitagliptin
Fasting blood glucose (mg/dL)					
Week (1)	97.8 ± 5.9	303.6 ± 117.9^a^	195.7 ± 39.8^a,b^	131.75 ± 18.9^b,c^	129.4 ± 34.1^b,c^
Week (2)	99.3 ± 5.9	369.8 ± 73.2^a^	96.75 ± 8.4^b^	109.7 ± 19^b^	131.7 ± 38.7^b,c^
Week (3)	97.2 ± 4.9	353.9 ± 48.6^a^	94.2 ± 9.9^b^	88.5 ± 11.3^b^	123.3 ± 27.3^a,b,c,d^
Week (4)	95.7 ± 5.5	285.1 ± 51.4^a^	98.5 ± 6.7^b^	87.9 ± 13.5^b^	108.5 ± 14.6^b^

Serum insulin (*µ*IU/mL) at the end of the treatment period	1.8 ± 0.3	11.5 ± 0.6^a^	7.27 ± 0.5^a,b^	9.78 ± 0.5^a,b,c^	10.1 ± 0.7^a,b,c^

HOMA-IR at the end of the treatment period	0.43 ± 0.1	8.1 ± 1.4^a^	1.8 ± 0.1^a,b^	2.1 ± 0.3^a,b^	2.7 ± 0.4^a,b,c^

Values are presented as mean ± SD (*n* = 10). ^a^Significantly different from the control group, ^b^significantly different from the diabetic untreated group, ^c^significantly different from the Metformin group, and ^d^significantly different from the Glimepiride group, using ANOVA (LSD), *P* value < 0.05.

## Abbreviations

T2DM: Type 2 diabetes mellitus
NEFA: Nonesterified fatty acids
HFD: High-fat diet
STZ: Streptozotocin
DPP-4: Dipeptidyl peptidase-4
GIP: Glucose-dependent insulinotropic peptide
GLP-1: Glucagon-like peptide-1
FBG: Fasting blood glucose
DMSO: Dimethyl sulfoxide
OGTT: Oral glucose tolerance test
IRI: Insulin resistance index
HOMA-IR: Homeostasis model assessment estimate of insulin resistance
ELISA: Enzyme-linked immunosorbent assay
ANOVA: Analysis of variance
LDL-C: Low density lipoprotein-cholesterol
HDL-C: High density lipoprotein-cholesterol
AMPK: AMP-activated protein kinase
SUR1: Sulphonylurea receptors 1.
